# ﻿Morphological diversity of pistil stigmas and its taxonomic significance of representatives of holoparasitic Orobanchaceae from Central Europe

**DOI:** 10.3897/phytokeys.215.96263

**Published:** 2022-12-06

**Authors:** Karolina Ruraż, Renata Piwowarczyk

**Affiliations:** 1 Center for Research and Conservation of Biodiversity, Department of Environmental Biology, Institute of Biology, Jan Kochanowski University, Uniwersytecka 7, PL-25-406, Kielce, Poland Jan Kochanowski University Kielce Poland

**Keywords:** flower morphology, gynoecium, *Orobanche*, *Phelipanche*, taxonomy

## Abstract

The stigma is the terminal part of the carpel which receives pollen during the pollination process. Although the role of the stigmas in pollination is the same for all angiosperms, stigmas structures are very diverse. This study aimed to evaluate intraspecific, interspecific and intergeneric stigmas variability and then find differences of the stigma morphology amongst 24 holoparasitic *Orobanche* and *Phelipanche* species and provide new insights into its potential taxonomic value. This paper was also focused on selecting the best diagnostic features that would be used for future stigma analysis in other species of Orobanchaceae. These analyses were conducted with fresh, dry and fixed material using stereomicroscopy from different locations from Central Europe. Twenty-one quantitative or qualitative morphological features were analysed. This study highlights the variation of stigma morphology and characters which are useful to improve the taxonomic understanding of problematic taxa. Thus, two main types of stigmas were established, based on tested features: 1–oval, rarely hemispherical in shape, most often one-coloured with lobes separated in *Phelipanche* stigmas; 2–spherical to hemispherical, rarely oval, multi-coloured with partially fused or separated lobes in *Orobanche* stigmas. The best diagnostic features of the stigmas for distinguishing the Orobanchaceae are the type and subtype of stigma, the length and area of the stigma, the width of single lobes, the width in the middle part of the stigma, the length of upper and lower separation in the middle part between lobes and the angle between lobes in the upper and lower part. The morphological features of the stigmas are important criteria for distinguishing genera, sections and subsections, as well as related species. In this study, we present the first stigma morphological studies for the most numerous genera from the tribe Orobancheae and this paper may determine features possible to use in solving certain taxonomic problems and evolutionary relationships of the species.

## ﻿Introduction

Orobanchaceae is the largest parasitic plant family with 102 genera and over 2,100 species (Nickrent 2020). In this family, the tribe Orobancheae is the oldest and most species-rich lineage of holoparasitic Orobanchaceae (McNeal et al. 2013; Schneider and Moore 2017; Piwowarczyk et al. 2021). Due to the strongly reduced vegetative organs of obligate parasitic plants, they belong to the most critical genera of world flora and cause many difficulties and mistakes in species identification. They do not form vegetative organs such as leaves (reduced to simple scales) and their appearance is limited to generative stems with flowers, highly variable in colour and morphology, so any additional characteristics of systematic value would be helpful.

Recently, studies on the molecular phylogeny and taxonomy of Orobanchaceae have clarified many controversial issues (Piwowarczyk et al. 2017, 2018, 2019, 2021 and references therein). The relationships within several important lineages can also be explained by additional morphological analysis. Previous studies on holoparasitic Orobanchaceae seeds and pollen morphology (e.g. Abu Sbaih and Jury 1994; Abu Sbaih et al. 1994; Plaza et al. 2004; Domina and Colombo 2005; Zare et al. 2013; Piwowarczyk 2015; Piwowarczyk et al. 2015, 2016; Zare and Dönmez 2016; Piwowarczyk et al. 2020 and references therein) and petal micromorphology (Piwowarczyk and Kasińska 2017), as well as floral volatile organic compounds (VOC) (Tóth et al. 2016) have proved to be useful as significant complementary sources of taxonomic data.

The stigma is a part of the gynoecium, the female reproductive system of a flower, with an ephemeral and receptive surface of the style that collects the pollen and creates appropriate conditions for its hydration and germination. These female tissues also promote outcrossing or self-fertilisation by the coordinated timing of their maturation with pollen release (Heslop-Harrison 1981; Edlund et al. 2004). Understanding floral morphology is fundamental to evaluating the interactions between pollen grains and the stigmas, as well as for understanding the relationship between flowers and pollinators. Moreover, many species can only be identified by their floral characteristics (Heslop-Harrison 1992). Angiosperm stigmas are structurally very diverse and some families have stigmas of more than one type. However, stigma morphology can be used, similarly to pollen morphology, to assist taxonomic classifications (Heslop-Harrison and Shivanna 1977). The taxonomic importance and the variability of stigmas have been described in both monocotyledonous and dicotyledonous plants at the inter- and intrageneric level in for example, Bromeliaceae (Brown and Gilmartin 1989), Boragineae (Bigazzi and Selvi 2000), Vochysiaceae (Carmo-Oliveira and Morretes 2009), Convolvulaceae (Wright et al. 2011) and Cactaceae (Mosti et al. 2013).

Amongst the Orobanchaceae studied by Heslop-Harrison and Shivanna (1977), *Orobanche* s.l. presented a dry stigma with unicellular papillae. This group with dry stigmas was characterised by lack of any surface secretion and the subgroups separate into species, based on the presence of trichomes or papillae. A review of available literature showed a scarcity of data describing the morphological variation of Orobancheae stigmas. Teryokhin et al. (1993) studied the morphological differences of the stigmas for about 50 taxa of the *Orobanche* L. and *Phelipanche* Pomel genera. The stigmas of the tribe Orobancheae show considerable variation, i.e. often subglobose in *Cistanche* Hoffmannsegg & Link and usually discoid to subglobose in *Phelypaea* L. In the *Orobanche* and *Phelipanche* genera, the stigmas are 2–4-lobed with varied shape lobes, for example, elongated, spherical, ovate and/or flattened (Kreutz 1995; Teryokhin 1997; Piwowarczyk et al. 2019). Papers on the morphological descriptions of Orobancheae species have focused mainly on the colour and the degree of fusion of the stigma lobes (e.g. Kreutz 1995; Piwowarczyk et al. 2019; Thorogood and Rumsey 2021). Therefore, studies are necessary on the morphological variation and taxonomic significance of stigmas, especially in the case of systematic division or problematic groups of species. In Orobanchaceae, *Phelipanche* and *Orobanche* s. str. are the largest holoparasitic genera that comprise ca. 50–62 and 150 species, respectively (Piwowarczyk et al. 2019), which are parasitic on the roots of other vascular plants. These genera are characterised by worldwide distribution, especially in the Mediterranean Basin, western and central Asia, north Africa, while less represented in America and Oceania (alien) (Piwowarczyk et al. 2019, 2021). In this paper, we focused on the Central European area, with only five species in *Phelipanche*, in contrast to western and central Asia and the Mediterranean, where the genus *Orobanche* represents the largest genus with about 20–23 representatives (Piwowarczyk et al. 2018).

The present study aimed to evaluate intraspecific, interspecific and intergeneric stigmas variability and then find differences of stigma morphology representatives of the *Orobanche* and *Phelipanche* genera from Central Europe using stereomicroscopy and to provide new insights into its potential taxonomic value. One of the primary objectives was to describe the stigma morphology and identify stigma characters, based on qualitative and quantitative data and to evaluate how useful these characteristics are in systematics and diagnostics for the investigated taxa. This paper also focused on selecting the best diagnostic features that could be used for future stigma analysis in the remaining genera and species of Orobanchaceae.

## ﻿Materials and methods

### ﻿Plant material

Specimens and samples of stigmas for the present study were observed, collected and photographed by the authors (primarily by Piwowarczyk), mainly during several field trips between 2006 and 2022 in Central Europe (especially Poland, Czech Republic, Slovakia and Austria) and some complementary specimens were also observed from other parts of Europe. A total of 40 samples representing 24 species were analysed (5 *Phelipanche* and 19 *Orobanche*), usually from two different localities per species (Table 1). The study was based on fresh and mature specimens collected in the natural habitats and on dry herbarium specimens where mature stigmas were selected from herbarium collections, as well as on plant material which was fixed in FAA (Formalin-Aceto-Alcohol) solution. The fresh specimens were kept in the refrigerator, observed and recorded quickly to avoid dehydration within tens of minutes. Dried flowers, removed from herbarium specimens, were heated to boiling point and left to observe after several minutes. For the purpose of comparison and to eliminate variation that might be caused by sampling from different flower areas, we took mature stigmas close to the middle portion of an inflorescence. In addition, our observations and measurements of stigmas were also compared with numerous photographs of analysed species, as well as with problematic sections, subsections and/or series of species from other parts of Europe and Asia. Our samples have also been presented in recent molecular phylogenetic studies (Piwowarczyk et al. 2018, 2021). The plant names were updated, based on the World Flora Online (WFO) (2022), as well as the Index of Orobanchaceae (Sánchez Pedraja et al. 2016). Vouchers of plant material were deposited in the Herbarium (KTC) of the Institute of Biology, Jan Kochanowski University in Kielce (KTC acronym, according to Thiers 2017). Voucher information and geographic origin are included in Table 1. The terminology of stigma morphology was given according to Heslop-Harrison and Shivanna (1977), Heslop-Harrison (1992), Teryokhin et al. (1993), Kreutz (1995), Teryokhin (1997), Williams (2009), Wright et al. (2011) and Konarska and Chmielewski (2020). Systematic division was adopted according to Beck (1930) and Teryokhin et al. (1993), the scheme followed, explicitly or implicitly, by most researchers and some recent taxonomic changes (McNeill et al. 2012; Piwowarczyk et al. 2017, 2018, 2021).

**Table 1.** Species used in the present study and voucher information.

No	Species	Voucher	Host
1a	*Orobanchealba* Stephan ex Willd.	Poland, Kąty II, 15 July 2006, *R. Piwowarczyk* (KTC)	*Salviaverticillata* L.
1b	*O.alba*	Poland, Lasocin, 27 July 2006, *R. Piwowarczyk* (KTC)	*S.verticillata*
2a	*O.alsatica* Kirschl.	Poland, Kielce, Grabina Mt., 19 June 2021, *R. Piwowarczyk & K. Ruraż* (KTC)	*Peucedanumcervaria* (L.) Lapeyr.
2b	*O.alsatica*	Poland, Kąty near Zamość, 15 July 2006, *R. Piwowarczyk* (KTC)	*P.cervaria*
3	*O.artemisiae-campestris* Gaudin	Czech Republic, Mikulov, 21 June 2014, *R. Piwowarczyk* (KTC)	*Artemisiacampestris* L.
4a	*O.bartlingii* Griseb.	Poland, Podzamcze, 22 June 2021, *R. Piwowarczyk & K. Ruraż* (KTC)	*Seselilibanotis* W.D.J.Koch
4b	*O.bartlingii*	Poland, Cząstków, 30 June 2006, *R. Piwowarczyk* (KTC)	*S.libanotis*
5a	*O.caryophyllacea* Sm.	Poland, Kików, 28 May 2021, *R. Piwowarczyk & K. Ruraż* (KTC)	*Galiumverum* L.
5b	*O.caryophyllacea*	Poland, Łagiewniki, 13 June 2022, *R. Piwowarczyk* (KTC)	*G.mollugo* L.
6a	*O.coerulescens* Stephan in Willd.	Poland, Pasturka, 28 June 2022, *R. Piwowarczyk & K. Ruraż* (KTC)	*A.campestris*
6b	*O.coerulescens*	Poland, Dobrowoda, 19 June 2010, *R. Piwowarczyk* (KTC)	*A.campestris*
7	*O.cumana* Wallr.	Ukraine, Kherson, 31 May 2019, *R. Piwowarczyk* (KTC)	*Artemisia* sp.
8a	*O.elatior* Sutton	Poland, Dzierżysław near Kietrz, 11 July 2010, *R. Piwowarczyk* (KTC)	*Centaureascabiosa* L.
8b	*O.elatior*	Poland, Baldram, 10 July 2010, *R. Piwowarczyk* (KTC)	*C.scabiosa*
9a	*O.flava* Mart. ex F.W. Schultz	Poland, Tatra Mts, Mała Łąka Valley, 25 July 2014, *R. Piwowarczyk* (KTC)	*Petasiteskablikianus* Tausch ex Bercht.
9b	*O.flava*	Slovakia, Nizkie Tatra Mts, Ohniste, 4 August 2011, *R. Piwowarczyk* (KTC)	*P.kablikianus*
10	*O.gracilis* Sm.	Austria, Hundsheim, 21 June 2014, *R. Piwowarczyk* (KTC)	*Anthyllisvulneraria* L., Dorycniumpentaphyllumsubsp.germanicum (Gremli) Gams
11	*O.hederae* Duby	Spain, Elx, palm garden, 28 April 2009, *R. Piwowarczyk* (KTC)	*Hederahelix* L.
12a	*O.kochii* F.W. Schultz (=*O.centaurina* Bertol.)	Poland, Boria, 4 July 2021, *R. Piwowarczyk* (KTC)	*C.scabiosa*
12b	*O.kochii*	Poland, Pęczelice, Ostra Mt., 7 July 2022, *R. Piwowarczyk & K. Ruraż* (KTC)	*C.scabiosa*
13	*O.lucorum* A. Braun ex F.W. Schultz	Poland, Warsaw, Botanical Garden, 10 July 2009, *R. Piwowarczyk* (KTC)	*Berberisvulgaris* L.
14a	*O.lutea* Baumg.	Poland, Pęczelice, 28 May 2021, *R. Piwowarczyk & K. Ruraż* (KTC)	*Medicagofalcata* L.
14b	*O.lutea*	Poland, Ząbkowice, 29 May 2021, *R. Piwowarczyk & K. Ruraż* (KTC)	*M.sativa* L.
15a	*O.mayeri* (Suess. & Ronniger) Bertsch & F. Bertsch	Poland, Pieniny Mts, Trzy Korony, 30 July 2009, *R. Piwowarczyk* (KTC)	*Laserpitiumlatifolium* L.
15b	*O.mayeri*	Poland, Pieniny Mts, Białe Skałki, 29 July 2009, *R. Piwowarczyk* (KTC)	*L.latifolium*
16	*O.minor* Sm.	Poland, Żywiec, 19 July 2009, *R. Piwowarczyk* (KTC)	*Trifoliumrepens* L.
17a	*O.picridis* F. W. Schultz	Poland, Pińczów, 28 June 2022, *R. Piwowarczyk & K. Ruraż* (KTC)	*Picrishieracioides* L.
17b	*O.picridis*	Poland, Pęczelice, Ostra Mt., 7 July 2022, *R. Piwowarczyk & K. Ruraż* (KTC)	*P.hieracioides*
18a	*O.reticulata* Wallr.	Lubiatowo, June 2014, *R. Piwowarczyk* (KTC)	*Cirsiumarvense* (L.) Scop.
18b	*O.reticulata*	Slovakia, Nizkie Tatra Mts, Ohiste, 4 August 2011, *R. Piwowarczyk* (KTC)	*Carduusdefloratus* L.
19	*O.teucrii* Holandre	Austria, Hundsheim, 20 June 2014, *R. Piwowarczyk* (KTC)	*Teucriummontanum* L.
20a	*Phelipanchearenaria* (Borkh.) Pomel	Czech Republic, Mikulov, 21 June 2014, *R. Piwowarczyk* (KTC)	*A.campestris*
20b	*P.arenaria*	Poland, Młyny, 28 June 2022, *R. Piwowarczyk & K. Ruraż* (KTC)	*A.campestris*
20c	*P.arenaria*	Poland, Pasturka, 28 June 2022, *R. Piwowarczyk & K. Ruraż* (KTC)	*A.campestris*
20d	*P.arenaria*	Poland, Zwierzyniec, 29 June 2021, *R. Piwowarczyk & K. Ruraż* (KTC)	*A.campestris*
21	*P.bohemica* (Čelak.) Holub	Poland, Zawiercie, 11 July 2010, *R. Piwowarczyk* (KTC)	*A.campestris*
22	*P.caesia* (Rchb.) Soják	Ukraine, Askania Nova, 16 June 2011, *R. Piwowarczyk* (KTC)	*A.austriaca* Jacq.
23	*P.purpurea* (Jacq.) Soják	Poland, Chrzanów, 18 June 2009, *R. Piwowarczyk* (KTC)	*Achilleamillefolium* L.
24a	*P.ramosa* (L.) Pomel	Poland, Brzeziny, 4 September 2021, *R. Piwowarczyk & K. Ruraż* (KTC)	*Nicotianatabacum* L.
24b	*P.ramosa*	Poland, Szewce, 15 September 2013, *R. Piwowarczyk* (KTC)	*Solanumlycopersicum* L.

### ﻿Morphometric analysis

**Figure 1.**
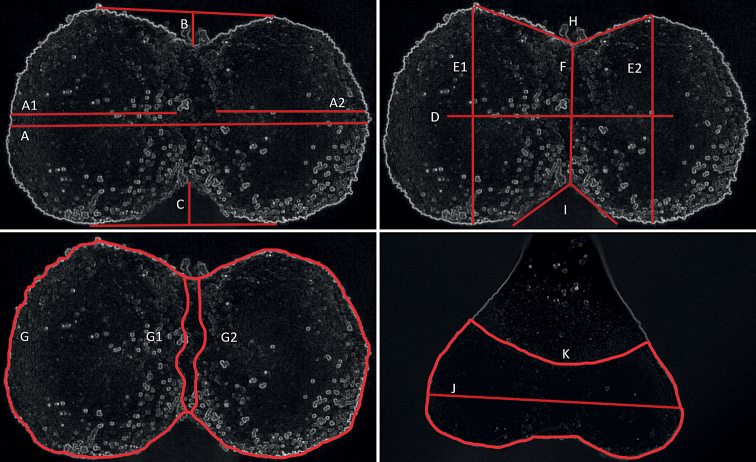
Measurement scheme of a 2-lobed stigma of *Orobanche*. **A–I** bottom view **J, K** front view **A** the length of 2-lobed stigma (µm) **A1, A2** the length of single lobes (µm) **B** the length of upper separation in the middle part between lobes (µm) **C** the length of lower separation in the middle part between lobes (µm) **D** the length of the mouth of the stylar canal (slit) (µm) **E1, E2** the width of single lobes (µm) **F** the width in the middle part of the stigma (µm) **G** the area of 2-lobed stigma (µm^2^) **G1, G2** the area of single lobes (µm^2^) **H** the angle between 2-lobed stigma in the upper part (°) **I** the angle between 2-lobed stigma in the lower part (°) **J** the length of 2-lobed stigma (µm) **K** the area of 2-lobed stigma (µm^2^).

Twenty-one quantitative and qualitative morphological features were measured. Sixteen quantitative features were analysed in the bottom view, i.e. the length of 2-lobed stigma (typical) (A) (µm), the length of single lobes (A1, A2) (µm), the length of upper separation in the middle part between lobes (B) (µm), the length of lower separation in the middle part between lobes (C) (µm), the length of the mouth of the stylar canal (slit) (D) (µm), the width of single lobes (E1, E2) (µm), the width in the middle part of the stigma (F) (µm), the area of 2-lobed stigma (G) (µm^2^), the area of single lobes (G1, G2) (µm^2^), the angle between 2-lobed stigma in the upper part (H) (°) and the angle between 2-lobed stigma in the lower part (I) (°). In the front view, two morphological features were examined, i.e. the length of 2-lobed stigma (J) (µm) and the area of 2-lobed stigma (µm^2^) (K) (Fig. 1). Additionally, five qualitative features, namely type, subtype, shape (in bottom view), colour and the degree of stigma lobes separation were taken into account for the morphological analysis of the stigmas.

Morphological observations of the stigmas were carried out using a Nikon SMZ-800 stereoscopic microscope coupled with a NIKON DSFi3 camera (Tokyo, Japan). Measurements were made using AxioVision SE64 Rel. 4.9.1 software (Carl Zeiss, Germany). For morphological characterisation, 30–50 stigmas of mature flowers from 5–10 randomly selected individuals of each sample of species were used. Data analyses were performed using Statistica 13 (TIBCO Software Inc. 2017). Eleven quantitative and two qualitative characters of stigmas were analysed using UPGMA, i.e. the length of 2-lobed stigma (typical) (A) (µm), the length of upper separation in the middle part between lobes (B) (µm), the length of lower separation in the middle part between lobes (C) (µm), the width of single lobes (E1, E2) (µm), the width in the middle part of the stigma (F) (µm), the area of 2-lobed stigma (G) (µm^2^), the angle between 2-lobed stigma in the upper part (H) (°), the angle between 2-lobed stigma in the lower part (I) (°), the length of 2-lobed stigma (J) (µm) and the area of 2-lobed stigma (µm^2^) (K), as well as the type and subtype of the stigma. These features were chosen because they showed the differences and similarities between species. A dendrogram was prepared, based on the similarity matrix generated using Gower’s general similarity coefficient (Gower 1966). Both analyses were performed using the MVSP package version 3.1 (Kovach 1999).

## ﻿Results

### ﻿General characteristics of stigma

**Figure 2.**
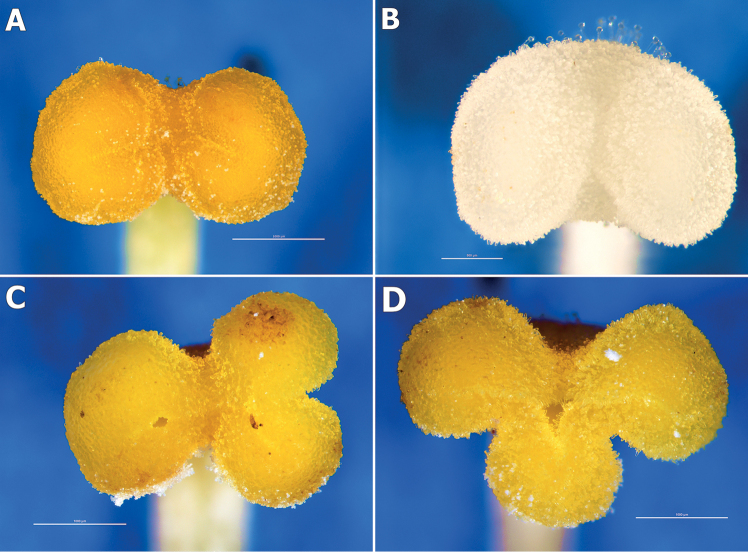
Micrographs of typical 2-lobed stigmas of *Orobanche* and *Phelipanche* and occasional 3-lobed stigmas of *Orobanche*. **A***Orobanche***B***Phelipanche***C, D** 3-lobed stigmas of *Orobanche*. Scale bars: 1000 µm (**A, C, D**); 500 µm (**B**). Phot. K. Ruraż, J. Posłowska and K. Zubek.

Morphological characterisation of the stigmas of *Orobanche* and *Phelipanche* has provided important data for the taxonomy of Orobancheae. The study showed some morphological similarities in stigma characters in both genera, for example, stigma usually 2-lobed, occasionally 3- and 4-lobed, lobes spherical to ovulate in shape (Fig. 2). The third lobe may be centrally located between the two lobes or directly under one of the lobes (Figs 2C, D). The centrally narrowed stigma was bent towards the lower lip and in the middle part was described by the presence of the mouth of the stylar canal. The stigma was covered with numerous papillae concentrated on the coloured lobes that were regularly arranged. The bow-shaped stigma had a viscous substance on the surface at the receptive stage. In buds, the length of the mouth of the stylar canal (slit) was more visible because it did not contain this substance.

### ﻿*Orobanche*

**Figure 3.**
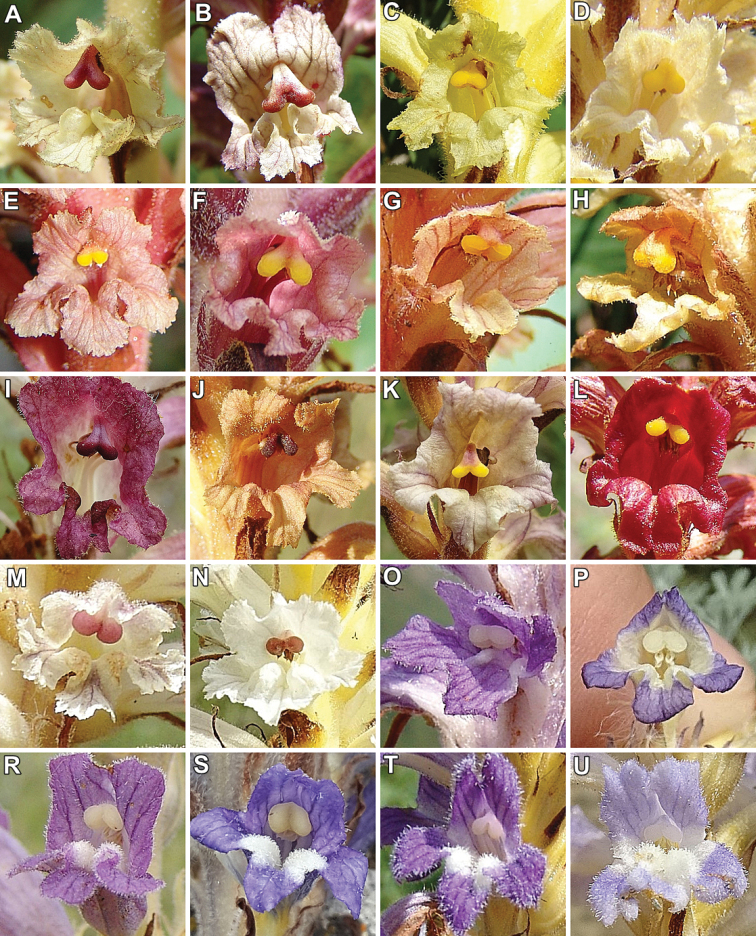
General habit of selecting flowers of Orobanchaceae species with a stigma in the front view. **A***Orobanchealba***B***O.reticulata***C***O.alsatica***D***O.bartlingii***E***O.kochii***F***O.elatior***G***O.flava***H***O.mayeri***I***O.caryophyllacea***J***O.teucrii***K***O.lutea***L***O.gracilis***M***O.minor***N***O.picridis***O***O.coerulescens***P***O.cumana***R***Phelipanchearenaria***S***P.caesia***T***P.purpurea***U***P.ramosa*. Phot. R. Piwowarczyk.

**Figure 4.**
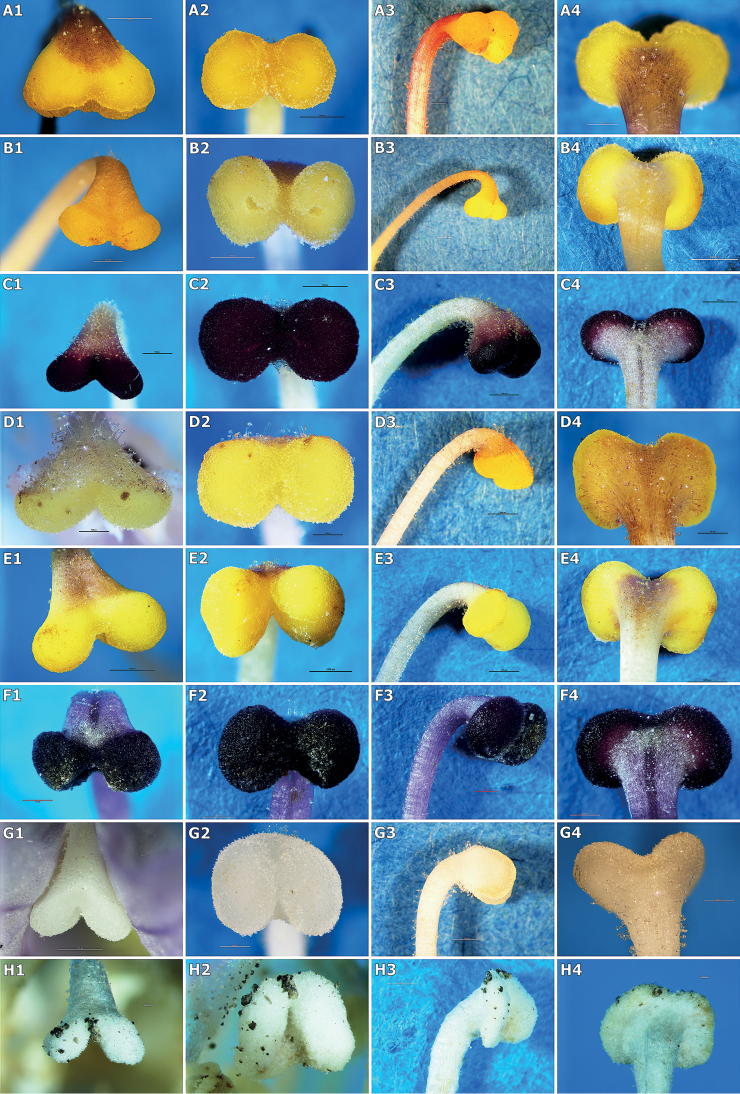
Micrographs of selected stigmas of the studied species. **A***Orobanchealsatica***B***O.bartlingii***C***O.caryophyllacea***D***O.kochii***E***O.lutea***F***O.picridis***G***Phelipanchearenaria***H***P.ramosa*. **1** front view **2** bottom view **3** lateral view **4** back view. Scale bars: 1000 µm (**A1–A3, B1–C4, D3, E1–E4, G1, G3**); 500 µm (**A4, D1, D2, D4, F1–F4, G2, G4, H3**); 100 µm (**H1, H2, H4**). Phot. K. Ruraż, J. Posłowska and K. Zubek.

The stigmas of *Orobanche* were hemispherical, spherical, rounded, rarely oval. These stigmas were varied in colour from white, yellow, orange, pink, purple, red to dark brown with partially fused or separated lobes (Table 2, Figs 2A, 3A–P, 4A–F). In the field research, two forms were observed in *Orobanche*, euchrome (normal colour) and hypochromic (yellow), which were easy to distinguish. We observed that the hypochromic form was smaller than the euchromic in each feature tested.

**Table 2.** The comparison of the stigmas of selected species of *Orobanche* and *Phelipanche*.

No	Species	The length of 2-lobed stigma (A) (µm)			The length of single lobes (A1) (µm)			The length of single lobes (A2) (µm)			The length of upper separation in the middle part between lobes (B) (µm)			The length of lower separation in the middle part between lobes (C) (µm)			The length of the mouth of the stylar canal (slit) (D) (µm)			The width of single lobes (E1) (µm)			The width of single lobes (E2) (µm)			The width in the middle part of the stigma (F) (µm)			The area of 2-lobed stigma (G) (µm^2^)			The area of single lobes (G1) (µm^2^)			The area of single lobes (G2) (µm^2^)			The angle between 2-lobed stigma in the upper part (H) (˚)			The angle between 2-lobed stigma in the lower part (I) (˚)			The length of 2-lobed stigma (J) (µm)			The area of 2-lobed stigma (µm^2^) (K)			Type	Subtype	Shape	Colour	Degree of stigma lobes separation
		Min	Mean	Max	Min	Mean	Max	Min	Mean	Max	Min	Mean	Max	Min	Mean	Max	Min	Mean	Max	Min	Mean	Max	Min	Mean	Max	Min	Mean	Max	Min	Mean	Max	Min	Mean	Max	Min	Mean	Max	Min	Mean	Max	Min	Mean	Max	Min	Mean	Max	Min	Mean	Max					
1a	*Orobanchealba*	2406	2733	2956	1042	1267	1398	1053	1291	1420	250	400	436	175	322	390	850	1230	1420	1123	1334	1520	950	1183	1326	556	600	635	2757642	2850613	3000677	1245823	1324428	1445236	1123574	1249113	1445834	100	159	168	100	125	138	2553	2725	2953	1645762	1751163	1863425	2	4	5	dark red or purple (rarely yellow or orange)	2
1b	*O.alba*	2654	2769	3096	1095	1292	1423	1111	1263	1452	243	411	452	200	310	350	876	1249	1390	1002	1367	1457	1136	1156	1383	503	547	620	2651752	2977262	3100111	1235426	1383402	1445783	1124735	1242331	1445238	120	154	160	112	124	135	2389	2560	2756	1594525	1725078	1852642	2	4	5	dark red or purple (rarely yellow or orange)	2
2a	*O.alsatica*	2770	3263	3376	1258	1389	1506	1253	1394	1524	150	320	353	380	503	563	1255	1513	2024	1518	1725	1882	1534	1763	2044	713	1029	1217	2949725	4223848	5073149	1350001	1895292	2049205	1351501	1825268	2211640	106	121	149	90	99	142	2497	3155	3409	2003320	3070327	3438793	2	7	8	yellow	4
2b	*O.alsatica*	2776	3283	3423	1280	1390	1574	1289	1468	1600	152	331	362	385	502	559	1302	1513	2083	1552	1726	1889	1542	1765	2022	723	1168	1227	2952681	4234639	5045267	1345856	1864142	2057257	1325423	1948092	2354521	110	119	152	95	98	140	2500	3119	3375	2052425	3072867	3524578	2	7	8	yellow	4
3	*O.artemisiae-campestris*	2343	2489	2635	1055	1112	1169	824	848	872	269	294	318	305	308	333	952	1200	1425	1315	1435	1655	1296	1436	1481	736	862	988	2632733	2961322	3289911	1346029	1494916	1643804	1163778	1289765	1415751	110	117	125	115	127	140	2458	2559	2715	1405555	1625555	2025555	2	5	6	pink, purple-brownish, reddish	1
4a	*O.bartlingii*	2908	3289	3525	1324	1435	1649	1322	1440	1598	156	377	560	492	643	932	1415	1759	2136	1639	1754	1974	1453	1802	2128	625	850	976	3179984	4461832	5803293	1549428	2254127	2458532	1581602	2208461	2859009	97	118	152	36	62	96	3070	3222	3743	2789644	3172420	3854744	2	7	8	yellow	4
4b	*O.bartlingii*	3122	3290	3452	1352	1395	1658	1222	1352	1485	157	363	580	482	596	935	1423	1668	2098	1652	1796	1985	1425	1782	2119	636	846	998	3175235	4265832	5795423	1484526	2095321	2394258	1535275	2092462	2852954	95	123	155	40	68	90	3100	3207	3658	2758452	3157156	3792542	2	7	8	yellow	4
5a	*O.caryophyllacea*	2625	3234	4135	1174	1420	1814	1107	1411	1833	163	263	397	248	423	769	1307	1735	2150	1321	1644	1805	1356	1689	1925	546	899	1122	2952876	4194741	5952683	1495078	2133317	2856202	1067414	2118599	2998225	108	134	148	93	116	148	2542	3173	4003	2214881	3103345	3929173	2	6	7	dark brown, purple or rarely yellow or orange	5
5b	*O.caryophyllacea*	2717	3240	3857	1193	1431	1666	1149	1423	1714	162	257	325	253	436	685	1322	1753	2221	1504	1612	1871	1197	1607	1920	702	887	1047	2235708	4133380	5403435	1788448	2094521	2647715	1463544	2091128	2558874	123	139	146	102	116	146	3085	3160	3576	2902541	3125328	3968729	2	6	7	dark brown, purple or rarely yellow or orange	5
6a	*O.coerulescens*	2045	2280	2602	862	988	1047	821	939	1011	172	240	272	175	250	399	729	888	1143	849	1257	1588	823	1307	1652	637	979	1200	1545257	2107260	2763040	536064	633490	733232	453078	688153	753521	133	141	151	112	122	143	1667	2019	2455	785340	1105242	1235242	2	2	3	white to yellowish-white, rarely bluish	3
6b	*O.coerulescens*	2044	2270	2636	821	900	1100	850	990	1112	180	250	280	178	240	301	740	890	1200	870	1200	1600	840	1310	1700	650	999	1210	1586422	2108452	2794224	552222	654222	745252	475525	702541	765421	138	150	160	125	120	152	1600	2000	2389	796655	1100055	1245522	2	2	3	white to yellowish-white, rarely bluish	3
7	*O.cumana*	2191	2299	2600	650	950	1040	800	980	1200	195	243	290	190	247	325	790	952	1212	888	1215	1650	860	1325	1725	690	999	1242	1725555	2249511	2800502	652224	802121	1052555	681242	855521	104855	140	158	171	130	122	160	1600	2124	2544	799222	1255250	1352420	2	2	3	white to yellowish-white, rarely bluish	3
8a	*O.elatior*	2211	2750	3022	902	1200	1425	980	1192	1422	150	254	380	150	280	360	1100	1302	1380	1400	1500	1630	1182	1450	1690	782	1020	1098	2452252	3182522	3852424	1252522	1620222	1882522	1258522	1605525	1952522	125	138	150	110	130	140	2345	2685	3089	1555252	1922525	2742252	2	7	8	yellow	4
8b	*O.elatior*	2279	2665	3002	1040	1182	1402	1055	1182	1423	182	256	460	228	293	378	1120	1305	1450	1405	1520	1650	1252	1420	1752	800	1025	1100	2575255	3152522	3825551	1425254	1622252	1982522	1182525	1625444	1982202	118	140	162	115	128	153	2300	2688	3099	1505557	1922442	2654444	2	7	8	yellow	4
9a	*O.flava*	2440	3052	3325	1161	1339	1520	1026	1313	1455	182	222	253	215	339	441	1133	1441	1567	987	1308	1441	1045	1383	1414	564	787	958	2177820	3201648	3542524	1125475	1510172	1791079	1104524	1474925	1785244	136	147	158	122	135	149	3113	3431	4002	2954225	3182604	3525452	2	7	8	yellow, rarely orange	4
9b	*O.flava*	2480	3143	3389	1200	1400	1543	1130	1380	1487	185	227	260	220	340	448	1150	1482	1579	995	1358	1482	1096	1399	1487	568	790	987	2198525	3235255	3575225	1142524	1545722	1800241	1132522	1502142	1802141	139	149	159	125	137	149	3131	3458	4025	2982201	3325214	3685224	2	7	8	yellow, rarely orange	4
10	*O.gracilis*	3200	4091	4522	1523	1777	1952	1452	1569	1852	290	300	315	520	555	575	1950	2130	2300	950	1031	1100	1000	1083	1120	590	650	720	3352752	3836796	4525252	1524586	1891079	2152354	1535752	1839782	2165242	115	120	129	120	142	150	3522	4145	4522	3952435	4722574	5054242	2	3	4	yellow lobes with a reddish base of the stigma to the style tip	5
11	*O.hederae*	2316	2457	2620	1021	1114	1350	1034	1152	1250	250	300	400	270	310	410	1100	1200	1420	1242	1473	1652	1288	1499	1700	818	881	1002	2722202	3049217	3952542	1259925	1418678	1602020	1252522	1421152	1625521	105	125	130	98	114	125	2225	2418	2701	1425256	1686525	1915252	2	5	6	yellow	1
12a	*O.kochii*	2133	2657	2957	878	1190	1361	965	1174	1355	146	241	371	145	281	352	1071	1227	1334	1370	1503	1626	1131	1402	1669	751	1005	1063	2273587	3119816	3638828	1173520	1569933	1787545	1190538	1526655	1847662	116	134	149	103	126	138	2300	2616	3013	1548757	1903199	2612049	2	7	8	yellow	4
12b	*O.kochii*	2271	2682	2986	1034	1160	1347	1024	1140	1368	174	242	455	226	292	373	1098	1238	1421	1376	1507	1646	1211	1445	1731	794	1002	1094	2397616	3135407	3657502	1324292	1501738	1917114	1043585	1500763	1906603	108	130	155	110	124	152	2282	2605	3058	1445172	1905557	2580093	2	7	8	yellow	4
13	*O.lucorum*	2289	2885	3158	1025	1205	1325	885	1210	1299	157	205	234	189	302	407	995	1301	1358	887	1184	1275	884	1170	1247	480	687	862	2085214	3098547	3352524	1052442	1395241	1675252	1042252	1395241	1685221	128	140	148	115	129	141	2958	3258	3842	2752514	2958541	3295255	2	7	8	yellow	4
14a	*O.lutea*	2414	3213	3425	978	1251	1587	1124	1244	1509	207	261	321	313	434	555	1057	1406	1745	1368	1608	2031	1342	1624	1939	816	999	1232	3079257	4167377	5275009	1413468	2076596	2643138	1599790	2080971	2590285	116	133	144	78	119	135	2603	3075	3198	2506590	3062143	3355113	2	6	7	yellow, rarely orange	5
14b	*O.lutea*	2631	3252	3489	1158	1375	1721	1159	1358	1523	229	261	440	332	432	875	1091	1461	1869	1512	1675	2125	1616	1654	2010	820	989	1337	3134725	4177720	5565740	1540919	2167075	3080438	1607626	2150782	2816204	121	134	152	103	119	137	2690	3043	3363	2800458	3083820	3481223	2	6	7	yellow, rarely orange	5
15a	*O.mayeri*	2367	3024	3214	1124	1330	1501	1015	1301	1424	179	220	249	200	328	420	1086	1375	1499	979	1289	1399	999	1299	1400	524	775	942	2154424	3195242	3495521	1115471	1492201	1720214	1094254	1452142	1755288	129	138	147	120	130	145	3107	3401	3952	2884252	3125415	3452542	2	7	8	yellow	4
15b	*O.mayeri*	2380	2999	3203	1100	1289	1498	999	1299	1387	168	215	245	198	319	415	1079	1365	1487	968	1276	1384	990	1257	1397	517	768	935	2150254	3185143	3472514	1117524	1485214	1745241	1092514	1442252	1739541	130	142	149	119	132	144	3095	3398	3925	2895310	3099604	3385145	2	7	8	yellow	4
16	*O.minor*	2027	2333	2632	813	999	1113	804	987	1195	213	253	329	261	299	361	1023	1204	1423	1121	1315	1505	1176	1303	1596	577	737	923	1875234	2524575	3278563	956424	1240557	1575224	974255	1234452	1654224	109	124	139	95	114	119	2324	2499	2799	1425775	1635554	1835447	2	5	6	pinkish, reddish or purplish rarely white	1
17a	*O.picridis*	2039	2341	2748	796	996	1150	864	1029	1217	233	277	318	270	308	370	1175	1244	1480	1187	1372	1541	1200	1349	1470	732	800	952	2020061	2562134	3416204	904198	1264524	1627401	968520	1284620	1677785	107	123	132	98	114	121	2157	2525	2760	1420156	1687767	1853354	2	5	6	purple, dark red and pink	1
17b	*O.picridis*	2035	2333	2774	824	1004	1207	814	993	1208	249	273	339	270	292	371	1056	1201	1448	1157	1335	1542	1181	1339	1608	543	746	935	1890329	2539792	3313251	980526	1261885	1653207	992278	1250536	1731908	114	123	135	100	116	121	2392	2515	2890	1485242	1674790	1925422	2	5	6	purple, dark red and pink	1
18a	*O.reticulata*	2200	2459	2852	1050	1280	1400	1060	1270	1452	270	442	471	154	315	382	866	1249	1423	1245	1408	1555	995	1185	1324	570	603	645	2452534	2635921	2885422	1305424	1422813	1495242	1132531	1258456	1432574	123	150	161	92	122	140	2123	2582	2952	1782243	2023996	2174252	2	4	5	brownish or purplish as well as usually lighter in the upper part of the stigma	2
18b	*O.reticulata*	2300	2560	2962	1105	1315	1458	1110	1305	1482	275	445	478	156	320	385	876	1286	1487	1287	1458	1599	999	1198	1357	585	624	657	2475254	2668541	2895252	1312524	1432421	1502241	1272221	1475214	1562221	125	155	165	97	125	144	2157	2593	2999	1824224	2162437	2302552	2	4	5	brownish or purplish as well as usually lighter in the upper part of the stigma	2
19	*O.teucrii*	2617	3259	3935	1166	1339	1779	1100	1326	1756	160	230	366	250	430	770	1250	1600	1950	1258	1671	1752	1295	1679	1885	560	885	1085	2923211	4188569	5325321	1402542	1922681	2795212	1402674	1954136	2752642	111	140	150	95	117	152	2354	3075	3885	2256413	3188246	3954254	2	6	7	dark brown, purple	5
20a	*Phelipanchearenaria*	1880	2123	2397	785	824	1086	699	712	1023	185	192	295	151	327	368	402	432	616	1210	1480	1802	1250	1458	1704	621	851	977	2489552	2669838	3547202	1220672	1311531	1807843	1039657	1287672	1523739	120	125	134	107	127	154	2000	2183	2288	1415852	1679817	1865272	1	1	1	white	4
20b	*P.arenaria*	1866	2117	2360	824	947	1087	787	904	1055	191	237	309	124	279	350	514	623	804	1178	1448	1750	1258	1484	1714	764	864	999	2143661	2590398	3836144	1057719	1226497	1520612	1052145	1291165	1589813	117	125	133	120	139	154	1954	2000	2235	1405527	1657854	1852727	1	1	1	white	4
20c	*P.arenaria*	1960	2158	2443	870	1048	1121	796	959	1130	202	301	345	148	326	362	436	552	617	1238	1667	1962	1414	1673	1779	851	1010	1237	2752426	3521283	4052127	1224027	1712603	2042492	1126734	1535574	1649937	106	126	120	103	119	152	1888	1940	2135	1385424	1635680	1814525	1	1	1	white	4
20d	*P.arenaria*	1858	2181	2314	847	982	1112	711	901	1033	201	250	310	110	304	344	485	570	669	1338	1661	1803	1288	1617	1737	830	950	1098	2652524	2951856	3524525	1096897	1303751	1679465	894185	1194634	1460135	112	120	128	120	120	156	1856	1936	2099	1395472	1636061	1825577	1	1	1	white	4
21	*P.bohemica*	2089	2250	2500	987	1000	1083	869	1005	1183	240	252	263	202	242	337	558	622	640	1452	1570	1700	1476	1558	1602	974	1020	1128	2825152	3129718	3352525	1152525	1385251	1539616	1152555	1395225	1532525	119	125	130	111	108	121	1958	1999	2102	1502252	1702555	1842522	1	1	1	yellow-white	4
22	*P.caesia*	2059	2188	2400	929	961	999	948	974	986	300	305	306	286	310	389	500	515	635	1500	1702	1800	1519	1610	1700	890	900	918	3118762	3156066	3222055	1322021	1481583	1746409	1382380	1400571	1565378	110	129	140	107	116	120	1920	2065	2255	1385242	1654332	1834555	1	1	1	white	4
23	*P.purpurea*	2056	2219	2312	865	966	980	859	991	1156	230	242	250	190	205	328	552	602	620	1435	1462	1684	1452	1512	1598	950	1015	1114	2602565	2818684	3030555	1001678	1293136	1495412	1004524	1277624	1357524	100	119	125	101	108	120	1923	1983	2025	1496874	1739657	1836524	1	1	1	white or pale blue or violet	4
24a	*P.ramosa*	1159	1520	1670	481	673	717	469	565	621	163	198	226	200	236	315	312	367	477	811	1023	1113	814	979	1099	413	555	696	991449	1400737	1679621	332470	591670	759460	329541	586839	765428	82	101	116	99	108	118	1052	1288	1412	365241	449118	589117	1	1	2	white or bright bluish, rarely yellowish	4
24b	*P.ramosa*	1269	1568	1700	446	708	754	485	623	645	169	220	264	206	277	317	327	423	486	947	1206	1274	823	1165	1274	510	661	766	996252	1647702	1893740	369303	755309	845729	365225	708890	849552	78	94	106	90	101	112	1110	1323	1500	394527	487236	575272	1	1	2	white or bright bluish, rarely yellowish	4

Explanations: stigma type: 1–oval, rarely hemispherical, most often white, rare light blue or violet and yellow and lobes separated; 2–spherical, hemispherical, rounded, rarely oval, multi-coloured: white, yellow, orange, pink, purple, red to dark brown with partially fused or separated lobes; stigma subtype (in bottom view): 1–oval, rarely hemispherical, most often white, rare light blue or violet and yellow, lobes separated; 2–hemispherical, rarely rounded, white to yellowish-white, rarely bluish, lobes partially separated; 3–hemispherical to rounded, lobes distinctly separated; 4–spherical to rounded, multi-coloured, lobes in closer proximity; 5–hemispherical, multi-coloured, lobes in closer proximity or even partially united; 6–hemispherical to oval, multi-coloured, lobes distinctly separated; 7–hemispherical rarely oval, yellow, lobes separated; stigma shape (in bottom view): 1–oval, rarely hemispherical; 2–oval to hemispherical; 3–hemispherical, rarely rounded; 4–hemispherical to rounded; 5–spherical to rounded; 6–hemispherical; 7–hemispherical to oval; 8–hemispherical, rarely oval; degree of stigma lobes separation (in front view):1–closer proximity or even partially united; 2–closer proximity; 3–partially separated; 4–separated; 5–distinctly separated.

**Figure 5.**
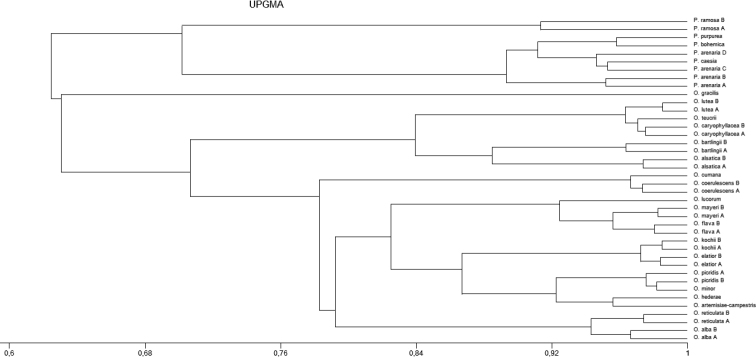
UPGMA dendrogram of morphological differentiation of Orobanchaceae stigmas.

**Figure 6.**
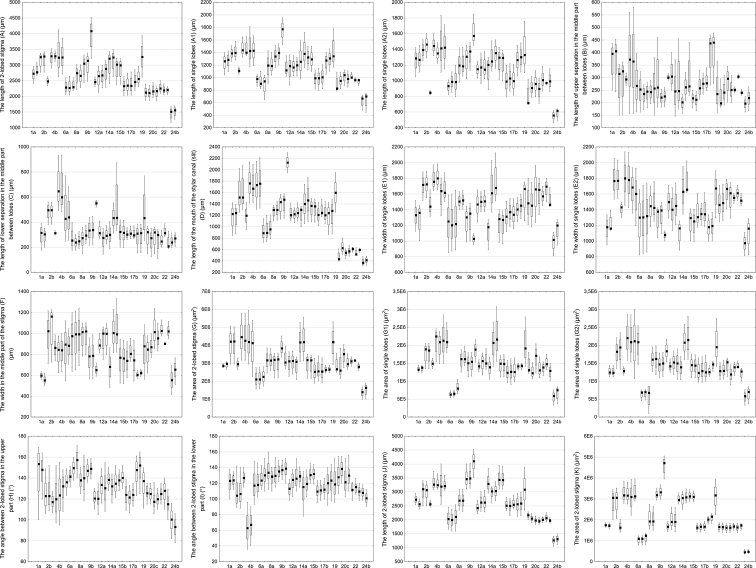
Box and whisker plots of quantitative morphological characters of Orobanchaceae stigmas. Points indicate the mean values (open square), boxes represent 25 and 75% percentiles and range (whiskers) represent 1 and 99% percentiles. Numbers indicate examined species (Table 2).

The stigmas of *O.alsatica*, *O.bartlingii*, *O.elatior*, *O.flava*, *O.kochii*, *O.lucorum* and *O.mayeri* belonging to the subsect. Curvatae (Beck) Piwow., Ó. Sánchez & Moreno Mor. consisted of two usually yellow lobes (in *O.flava* also orange lobes) which were hemispherical, rarely oval in shape and were separated (Table 2, Figs 3C–H, 4A, B, D). The UPGMA analysis on the basis of given features distinguished three subgroups: the first one included *O.alsatica* and *O.bartlingii*, the second was represented by *O.elatior* and *O.kochii* and the third by *O.flava*, *O.lucorum* and *O.mayeri* (Fig. 5). The analysed samples of *O.alsatica* and *O.bartlingii* were in a very close relationship and represented *O.alsatica* aggr. which is a problematic complex. In *O.bartlingii*, the shape of the mouth of the stylar canal (slit) was more irregular than *O.alsatica*. A visible difference in shape between species was marked between the lobes in the lower part and there was a larger separation in *O.bartlingii*. The stigmas in the front view in *O.bartlingii* were more pronounced and marked than in *O.alsatica*, which appear more flattened (Figs 4A1, B1). The length of 2-lobed stigma (A, J) of the first subgroup fell within a range of (2770–)3281(–3525) μm and (2497–)3176(–3743) μm, with an area (G, K) varying between (2949725–)4296538(–5803293) μm^2^ and (2003320–)3118193(–3854744) μm^2^. The width of single lobes (E1, E2) fell into a range of (1518–)1750(–1985) μm and (1425–)1778(–2128) μm, while the width in the middle part of the stigma (F) varied within a range of (625–)973(–1227) μm. The length of upper and lower separation in the middle part between lobes (B, C) was in a range of (150–)348(–580) μm and (380–)561(–935) μm and the angle between 2-lobed stigma in the upper and lower part (H, I), comprised (95–)120(–155)° and (36–)82(–142)° (Table 2, Fig. 6). In addition, the second and third subgroups were more similar, based on analysed features of stigmas to the previous one. The length of 2-lobed stigma (A, J) of the second subgroup (represented by *O.elatior* and *O.kochii*) comprised (2133–)2689(–3022) μm vs. (2289–)3021(–3389) and (2282–)2649(–3099) μm vs. (2958–)3389(–4025), with an area (G, K) equal to (2273587–)3147567(–3852424) μm^2^ vs. (2085214–)3183167(–3575225) μm^2^ and (1445172–)1913431(–2742252) μm^2^ vs. (2752514–)3138276(–3685224) μm^2^. In the second subgroup, the width of single lobes (E1, E2) varied between (1370–)1508(–1650) μm and (1131–)1429(–1752) μm and in the third (*O.flava*, *O.lucorum* and *O.mayeri*) varied in a range of (887–)1283(–1482) μm and (884–)1302(–1487) μm with the width in the middle part of the stigma (F) falling into a range of (751–)1013(–1100) μm vs. (480–)761(–987) μm. The length of upper and lower separation in the middle part between lobes (B, C) comprised (146–)248(–460) μm and (145–)287(–378) μm for *O.elatior* and *O.kochii* and (157–)218(–260) μm and (189–)323(–448) μm for *O.flava*, *O.lucorum* and *O.mayeri*. Finally, the angle between 2-lobed stigma in the upper and lower part (H, I) fell into a range of (108–)136(–162)° vs. (128–)143(–159)° and (103–)127(–153)° vs. (115–)133(–149)° (Table 2, Fig. 6).

*Orobanchecoerulescens* and *O.cumana* from the sect. Inflatae (subsect. Inflatae sensu Beck) consisted mostly of hemispherical, rarely rounded stigmas with two lobes which were white to yellowish-white, rarely bluish and partially separated (Table 2, Figs 3O, P). These species represented some of the smallest stigmas of the genus *Orobanche* that were analysed, i.e. (2044–)2283(–2636) μm and (1600–)2048(–2544) μm in the length of 2-lobed stigma (A, J) with an area (G, K) of (1545257–)2155074(–2800502) μm^2^ and (785340–)1153516(–1352420) μm^2^. The width of single lobes (E1, E2) comprised (849–)1224(–1650) μm and (823–)1314(–1725) μm, as well as the width in the middle part of the stigma (F) varied between (637–)992(–1242) μm. The length of upper and lower separation in the middle part between lobes (B, C) fell into a range of (172–)244(–290) μm and (175–)246(–399) μm and the angle between 2-lobed stigma in the upper and lower part (H, I) fell into a range of (133–)150(–171)° and (112–)121(–160)° (Table 2, Fig. 6).

*Orobanchegracilis* from the subsect. Cruentae Teryokhin had hemispherical to rounded and distinctly separated stigmas with two yellow lobes and with a reddish base of the stigmas to the style tip (Table 2, Fig. 3L). The length of 2-lobed stigma (A, J) was the largest of all Orobanchaceae stigmas tested, comprising (3200–)4091(–4522) μm and (3522–)4145(–4522) μm with an area (G, K) varying between (3352752–)3836796(–4525252) μm^2^ and (3952435–)4722574(–5054242) μm^2^. The width of single lobes (E1, E2) fell into a range of (950–)1031(–1100) μm and (1000–)1083(–1120) μm and the width in the middle part of the stigma (F) was equal to (590–)650(–720) μm. The length of upper and lower separation in the middle part between lobes (B, C) fell within a range of (290–)300(–315) μm and (520–)555(–575) μm and the angle between 2-lobed stigma in the upper and lower part (H, I) fell into a range of (115–)120(–129)° and (120–)142(–150)° (Table 2, Fig. 6).

*Orobanchecaryophyllacea*, *O.lutea* and *O.teucrii*, which are represented in the O.subsect.Orobanche (subsect. Galeatae sensu Teryokhin) had two hemispherical to oval and distinctly separated stigma lobes. The stigmas in *O.caryophyllacea* and *O.teucrii* had similar colours, namely dark brown, purple, rarely yellow or orange (in *O.caryophyllacea*), in contrast to *O.lutea* which were yellowish, rarely orange (Table 2, Figs 3I–K, 4C, E). *Orobanchecaryophyllacea* and *O.teucrii* stigmas were similar in terms of the analysis of qualitative and quantitative features, in contrast to *O.lutea* (Fig. 5). The length of 2-lobed stigma (A, J) of *O.caryophyllacea* and *O.teucrii* fell within a range of (2617–)3244(–4135) μm vs. (2414–)3233(–3489) and (2354–)3136(–4003) μm vs. (2603–)3059(–3363), with an area (G, K) varying between (2235708–)4172230(–5952683) μm^2^ vs. (3079257–)4172549(–5565740) μm^2^ and (2214881–)3138973(–3968729) μm^2^ vs. (2506590–)3072982(–3481223) μm^2^. In *O.caryophyllacea* and *O.teucrii*, the width of single lobes (E1, E2) comprised (1258–)1642(–1871) μm and (1197–)1658(–1925) μm, while in *O.lutea* displayed a range of (1368–)1642(–2125) μm and (1342–)1639(–2010) μm, with the width in the middle part of the stigma (F) in a range of (546–)890(–1122) μm vs. (816–)994(–1337) μm. The length of upper and lower separation in the middle part between lobes (B, C) was (160–)250(–397) μm and (248–)430(–770) μm for *O.caryophyllacea* and *O.teucrii* in comparison to (207–)261(–440) μm and (313–)433(–875) μm recorded in *O.lutea*. The angle between 2-lobed stigma in the upper and lower part (H, I) was equal to (108–)138(–150)° vs. (116–)134(–152)° and (93–)116(–152)° vs. (78–)119(–137)° (Table 2, Fig. 6).

The stigmas of species belonging to the subsect. Glandulosae (Beck) Teryokhin i.e. *O.alba* and *O.reticulata* consisted of two spherical to rounded lobes with different colours in closer proximity (Table 2, Figs 3A, B). *Orobanchereticulata* had more elongated and flattened stigmas with more separated lobes than *O.alba*, whose stigmas were dark red or purple (rarely yellow or orange), unlike *O.reticulata* which were brownish or purplish (mostly lighter in the upper part). The length of 2-lobed stigma (A, J) varied within limits of (2200–)2630(–3096) μm and (2123–)2615(–2999) μm with an area (G, K) falling within a range of (2452534–)2783084(–3100111) μm^2^ and (1594525–)1915669(–2302552) μm^2^. The width of single lobes (E1, E2) comprised (1002–)1392(–1599) μm and (950–)1181(–1383) μm, as well as the width in the middle part of the stigma (F) was equal to (503–)594(–657) μm. The length of upper and lower separation in the middle part between lobes (B, C) fell within a range of (243–)425(–478) μm and (154–)317(–390) μm and the angle between 2-lobed stigma in the upper and lower part (H, I) fell into a range of (100–)155(–168)° and (92–)124(–144)° (Table 2, Fig. 6).

Species from the subsect. Minores Teryokhin (incl. *O.hederae* from the subsect. Hederae Teryokhin) (*O.artemisiae-campestris*, *O.hederae*, *O.minor* and *O.picridis*) had two hemispherical lobes of the stigmas in closer proximity or even partially united. *Orobancheminor* stigmas varied in colour from pinkish, reddish or purplish to rarely white, similar to *O.artemisiae-campestris* which were pink, purple-brownish, reddish and *O.picridis* with purple, dark red and pink lobes and unlike *O.hederae* which were usually yellow. The distinguishing feature of the stigmas of this group was the presence of a well-developed and convex surface in relation to the base of the stigmas (Table 2, Figs 3M, N, 4F). The UPGMA analysis on the basis of given features distinguished two subgroups, i.e. the first one included *O.artemisiae-campestris* and *O.hederae* and the second was represented by *O.minor* and *O.picridis* (Fig. 5). The length of 2-lobed stigma (A, J) of the first subgroup was equal to (2316–)2473(–2635) μm vs. (2027–)2336(–2774) and (2225–)2489(–2715) μm vs. (2157–)2513(–2890), with an area (G, K) varying within limits of (2632733–)3005270(–3952542) μm^2^ vs. (1875234–)2542167(–3416204) μm^2^ and (1405555–)1656040(–2025555) μm^2^ vs. (1420156–)1666037(–1925422) μm^2^. In the first subgroup, the width of single lobes (E1, E2) varied between (1242–)1454(–1655) μm and (1288–)1468(–1700) μm and, in the second, varied in a range of (1121–)1341(–1542) μm and (1176–)1330(–1608) μm, with the width in the middle part of the stigma (F) in a range of (736–)872(–1002) μm vs. (543–)761(–952) μm. The length of upper and lower separation in the middle part between lobes (B, C) was (250–)297(–400) μm and (270–)309(–410) μm for *O.artemisiae-campestris* and *O.hederae* in comparison to (213–)268(–339) μm and (261–)300 (–371) μm recorded in *O.minor* and *O.picridis*. The angle between 2-lobed stigma in the upper and lower part (H, I) was in a range of (105–)121(–130)° vs. (107–)123(–139)° and (98–)121(–140)° vs. (95–)115(–121)° (Table 2, Fig. 6).

### ﻿*Phelipanche*

The stigmas were oval, rarely hemispherical, with separated lobes and were most often white, rarely light blue, violet and yellow (Table 2, Figs 2B, 3R–U, 4G, H). Stigmas are less varied and smaller in size than in *Orobanche*.

The stigmas of *Phelipanchearenaria*, *P.bohemica*, *P.caesia* and *P.purpurea* belonging to the sect. Trionychon (Wallr.) Piwow. & Ó. Sánchez (sect. Arenariae Teryokhin) consisted of two white lobes which were oval, rarely hemispherical with clearly separated lobes. Most often they were white, less often light blue or violet and yellow (Table 2, Figs 3R–T, 4G). Measurable data and UPGMA analysis showed the presence of two subgroups *P.arenaria* and *P.caesia* in the first, with the second represented by *P.bohemica* and *P.purpurea* (Fig. 5). The length of 2-lobed stigma (A, J) of the first subgroup comprised (1858–)2153(–2443) μm vs. (2056–)2235(–2500) and (1856–)2025(–2288) μm vs. (1923–)1991(–2102), with an area (G, K) of (2143661–)2977888(–4052127) μm^2^ vs. (2602565–)2974201(–3352525) μm^2^ and (1385242–)1652749(–1865272) μm^2^ vs. (1496874–)1721106(–1842522) μm^2^. In the first subgroup, the width of single lobes (E1, E2) fell within a range of (1178–)1592(–1962) μm and (1250–)1568(–1779) μm and, in the second, was equal to (1435–)1516(–1700) μm and (1452–)1535(–1602) μm, with the width in the middle part of the stigma (F) in a range of (621–)915(–1237) μm vs. (950–)1018(–1128) μm. The length of upper and lower separation in the middle part between lobes (B, C) was (185–)257(–345) μm and (110–)309(–389) μm for *P.arenaria* and *P.caesia*, compared to (230–)247(–263) μm and (190–)224(–337) μm recorded in *P.bohemica* and *P.purpurea*. The angle between 2-lobed stigma in the upper and lower part (H, I) fell into a range of (106–)125(–140)° vs. (100–)122(–130)° and (103–)124(–156)° vs. (101–)108(–121)° (Table 2, Fig. 6).

The stigmas of species belonging to the sect. Phelipanche i.e. *P.ramosa* was oval to hemispherical with separated lobes which were white or bright bluish, rarely yellowish (Table 2, Figs 3U, 4H). The length of 2-lobed stigma (A, J) was the smallest of all Orobanchaceae stigmas tested, comprising (1159–)1544(–1700) μm and (1052–)1306(–1500) μm with an area (G, K) of (991449–)1524220(–1893740) μm^2^ and (365241–)468177(–589117) μm^2^. The width of single lobes (E1, E2) fell into a range of (811–)1115(–1274) μm and (814–)1072(–1274) μm, as well as the width in the middle part of the stigma (F) was equal to (413–)608(–766) μm. The length of upper and lower separation in the middle part between lobes (B, C) fell within a range of (163–)209(–264) μm and (200–)257(–317) μm and the angle between 2-lobed stigma in the upper and lower part (H, I) fell into a range of (78–)98(–116)° and (90–)110(–118)° (Table 2, Fig. 6).

### ﻿Morphometric analysis

The analysis of data from the observation of stigmas structures identified twenty-one morphological characters of stigmas in Orobancheae (Table 2). UPGMA analysis based on characteristics suggested two types of stigma morphology, the first one included 2-lobed, occasionally 3- and 4-lobed stigmas which were oval, rarely hemispherical in shape and most often white, rarely light blue and yellowish with separated lobes. This cluster was represented by all *Phelipanche* species studied (Fig. 5). A separate subgroup consisted of *P.ramosa* stigmas characterised by being the smallest of all stigmas tested, as well as by their more hemispherical shaped stigmas in relation to other *Phelipanche*. The second type comprised the largest group of *Orobanche* species studied with 2-lobed, occasionally 3- and 4-lobed stigmas, mostly hemispherical to rounded or rarely oval and varied in colour from white, yellow, orange, pink, purple, red to dark brown with partially fused or separated lobes. This group was highly varied in terms of most useful identifying features, such as subtype, the length of 2-lobed stigma (A, J), the area of 2-lobed stigma (G, K), the width of single lobes (E1, E2), the width in the middle part of the stigma (F), the length of upper and lower separation in the middle part between lobes (B, C) and the angle between 2-lobed stigma in the upper and lower part (H, I). These features allowed several subgroups of stigmas to be distinguished corresponding to systematic and phylogenetic groups (Fig. 5). The first subgroup consisted of *O.gracilis* stigmas, which were the largest stigmas of all Orobanchaceae tested. *Orobanchecaryophyllacea*, *O.lutea* and *O.teucrii* stigmas created a subgroup that stands out from the rest, with hemispherical to oval stigmas and two distinctly separated stigma lobes. *O.alsatica* and *O.bartlingii* stigmas reached one of the largest sizes after *O.gracilis* (Table 2, Fig. 6). The most diverse group on the UPGMA was represented by stigmas of the species from the *Curvatae* subsection, where three subgroups were distinguished (Fig. 5). The first of them, consisting of the stigmas of *O.alsatica* and *O.bartlingii*, was further away from the other representatives of this subsection due to quantitative features, for example, these stigmas were larger in relation to the other representatives such as: *O.elatior*, *O.flava*, *O.kochii*, *O.lucorum* and *O.mayeri*. The next subgroup, corresponding to *O.coerulescens* and *O.cumana*, had the smallest stigmas of the genus *Orobanche* that were analysed with a characteristic colour from white to yellowish-white, rarely bluish. *Orobancheartemisiae-campestris*, *O.hederae*, *O.minor* and *O.picridis* stigmas formed a separate subgroup of small stigmas (on average up to 2500 μm in length) with lobes in closer proximity or even partially united. A distinctive feature of the stigmas of this subgroup was also the presence of a well-developed and convex surface in relation to the base of the stigmas. The last subgroup, *O.alba* and *O.reticulata*, had stigmas which consist of a specific shape from spherical to rounded with two lobes in closer proximity and reaching similar sizes (Fig. 5).

## ﻿Discussion

In order to provide diagnostic information and to evaluate the utility of stigma morphological characters considered in a taxonomic and phylogenetic context, a more thorough study on stigma morphology of representatives of Orobanchaceae was performed. For this purpose, we selected species representing various localities from Central Europe and applied stereomicroscopy to provide additional evidence for distinguishing genera, sections or subsections, as well as some related species of Orobancheae.

*Orobanche* and *Phelipanche* genera can be divided into two groups on the basis of the analysis of features carried out in this study. It was found that some quantitative features (e.g. the length of 2-lobed stigma (A, J), the area of 2-lobed stigma (G, K), the width of single lobes (E1, E2), the width in the middle part of the stigma (F), the length of upper and lower separation in the middle part between lobes (B, C), the angle between 2-lobed stigma in the upper and lower part (H, I) and the type and subtype of the stigma) are the best diagnostic characteristics for distinguishing these genera (Fig. 5). The stigmas in *Phelipanche* were the smallest of the species studied, usually not more than 2.5 mm in length of 2-lobed stigma (A) (Table 2, Fig. 6). There was a clear difference in the size of the stigmas between the analysed sections in *Phelipanche*. The stigmas of species belonging to the sect. Phelipanche i.e. *P.ramosa* were smaller (up to 1700 μm) than the species representing the sect. Trionychon (Arenariae) (the minimum length of 2-lobed stigma (A) was 1858 to 2089 μm) (Table 2, Fig. 6). Morphological, ecological and molecular differences suggest that *P.bohemica* have been a separate species (Piwowarczyk 2012, 2015; Piwowarczyk et al. 2015, 2018). Morphological studies of stigmas also showed differences between *P.purpurea* and *P.bohemica*, which had yellow-white stigmas in contrast to *P.purpurea* which was whitish (Table 2). Furthermore, the analysis of characteristics of stigmas confirmed the separation of two species of the problematic complex *O.alsatica* aggr. *Orobanchealsatica* and *O.bartlingii* were different in the shape and in the length of the mouth of the stylar canal (slit), which was more regular in *O.alsatica*, as well as there being a larger separation in the lower part between lobes in *O.bartlingii* (Figs 4A2, B2, 6). However, the similarity was evident in size, for example, both species were larger than the rest of the species in subsect. Curvatae. *Orobanchecoerulescens* and *O.cumana* (sect. Inflatae) have been a transitional position between *Orobanche* and *Phelipanche*. They were placed on phylogenetic trees outside the rest of *Orobanche* (Piwowarczyk et al. 2018, 2021), which correlated with some phenotypic features (such as the violet colour of the flowers and white stigmas) or tricolpate pollen (in *O.coerulescens*) (Piwowarczyk et al. 2015) that made them similar to *Phelipanche* species. Our studies confirmed that the stigma characteristics corresponded to *Orobanche*. However, based only on the colour of the stigmas, they were close to *Phelipanche* (Fig. 5). Species belonging to the section Minores (incl. subsect. Hederae) (*O.artemisiae-campestris*, *O.hederae*, *O.minor* and *O.picridis*) and the section Inflatae (*O.coerulescens* and *O.cumana*) were clearly distinguished from others on the basis of their length of 2-lobed stigma (A) (µm), usually not more than 3 mm in *Orobanche* (Table 2, Fig. 6). *O.hederae* (which is surprisingly regarded as a member of the *Inflatae* section, following Teryokhin et al. 1993) was clustered on phylogenetic trees with species belonging to the subsect. Minores (Piwowarczyk et al. 2018) and our stigma morphology (Fig. 5) supports the supposition that these species are relatives.

In addition, phylogenetic studies support seed and pollen micromorphological analysis and it is noteworthy that this study showed the separation of the subgroups into separate species, based on the morphological analysis of stigmas which corresponded to systematic and phylogenetic groups (Piwowarczyk et al. 2018, 2021). Consequently, ITS dendrograms and cluster analysis (UPGMA) were similar, for example, showing a clear difference between the morphology of the stigmas of *Orobanche* and *Phelipanche* (Fig. 5). In conclusion, the often well-defined features of the stigmas had value both as taxonomic characters and as phylogenetic data for systematic studies. Additionally, the stigmas of the pistil of other members of the tribe Orobancheae, i.e. *Cistanche* and *Phelypaea* genera, have not been thoroughly investigated morphologically. However, there are some papers describing their shape and colour. *Cistanche* species are often subglobose in shape and usually white, yellowish or bluish, while stigmas in *Phelypaea* are usually discoid to subglobose with red, pink, rarely yellow colours (e.g. Teryokhin 1997; Piwowarczyk et al. 2019).

Morphological analysis of the stigmas of Central European broomrapes showed that they were characterised by high variability at the intergeneric and interspecific level. The morphology of the stigmas has consistently provided additional data to the other characters of flower morphology used to separate species, i.e. the type and subtype of stigma, the length of 2-lobed stigma (A, J), the area of 2-lobed stigma (G, K), the width of single lobes (E1, E2), the width in the middle part of the stigma (F), the length of upper and lower separation in the middle part between lobes (B, C) and the angle between 2-lobed stigma in the upper and lower part (H, I) (Table 2). Interestingly, the available publications of observations of the shape of stigmas in the Orobanchaceae concerned mainly the analysis of this feature in the front view. However, as our research showed, the most useful features can be seen when observing the stigmas from the bottom side, which has not been studied before. The features studied allow us to distinguish between species taking into account both dry, as well as FAA solution material. Additionally, the length of single lobes (A1, A2) and the area of single lobes (G1, G2) could be helpful when using larger samples. Features such as shape, colour and the degree of stigma lobes separation also have diagnostic value and allow us to distinguish species and genera in Orobanchaceae (e.g. Kreutz 1995; Piwowarczyk et al. 2019; Thorogood and Rumsey 2021), based on these characters. Moreover, the colour of the stigmas and petals of the same individual were often contrasting and differ from each other. Therefore, it is important to observe fresh material because the colours of the flowers of holoparasitic species in the field turn to different shades of brown after drying.

Although the taxonomy of some Orobanchaceae is still controversial, the morphology of stigmas could provide the next important characters used to define species. In this study, we used fresh plant material, dried and fixed in FAA solution. According Heslop-Harrison (1992), the most informative images or morphological analysis of stigmas are obtained using fresh, unfixed and uncoated material, because methods where the material is processed are unnecessary and leave some artefacts. Furthermore, it is extremely important that the morphological features of the mature stigmas are observed and collected at the stage when the stigmas are receptive for pollination. Additionally, the use of a stereoscopic microscope in research on the morphology of stigmas of Orobanchaceae are another possibility for differentiating taxonomically problematic species. Teryokhin et al. (1993) analysed the morphological differences of the stigmas of about 50 taxa of *Orobanche* and *Phelipanche* genera. In this paper, several types of stigmas were mentioned, i.e. discoid, discoid-bilobed, bilobed, two-columned-discoid, as well as two-columned stigma. This division proposed by Teryokhin et al. (1993) concerned the analysis only of the shape of the stigmas and the degree of separation of the lobes. Unfortunately, taking only these features into account, we cannot come to a conclusion about the usefulness of stigmas’ features for the taxonomy of Orobanchaceae (Teryokhin et al. 1993), except on a general level. Our observations showed that the degree of stigma separation of a particular species varies depending on the stage of stigma development and was difficult to observe and measure and, therefore, this feature should not be taken into account in separate systematic considerations. In conclusion, the paper of Teryokhin et al. (1993) is the only work known to us that draws wider attention to the morphology of the stigmas of holoparasites from Orobancheae. According to Yang et al. (2002), a study of stigma features and other parts of flowers when considered together can allow a better understanding of the process of floral evolution of hemiparasitic *Pedicularis*, especially their significance in pollination adaptation.

Heslop-Harrison and Shivanna (1977) classified *Orobanche* s.l. stigmas as dry stigmas with unicellular papillae, but sometimes the secretion may appear under a detached surface cuticle when it has been damaged by pollinating insects. Interestingly, the morphological adaptations of the stigmas were critical for optimum capture of pollen grains. According to Heslop-Harrison and Shivanna (1977), trinucleate pollen tends to be associated with dry stigmas. However, binucleate pollen occurs with both wet and dry stigmas and, in the case of *Orobanche*, the pollen is binucleate and the stigmas are dry. However, the basic subdivision in some families (e.g. Onagraceae) has been problematic and, in some cases, no clear separation can be made (Heslop-Harrison 1981). Amongst angiosperms families, Orobanchaceae is distinguished by the diversity in stigma type, in contrast to most families, in which stigmas are homogeneous (Heslop-Harrison 1981).

## ﻿Conclusions

Comparative studies will be required to test further findings about the morphological determinants in stigmas of such variation in the Orobanchaceae family. The possibility of using the morphology of the stigmas may help explain taxonomic relationships in the identification of specimens of problematic taxa. Flowers, including stigmas of holoparasitic plants, have evolved several adaptations for pollination as a process of their parasitic strategies. In addition, floral characters have a special significance in the investigation of parasitic plants whose life cycle has led to a reduction of vegetative structures. A study of the floral stigmas may be useful to systematics and to obtaining knowledge of ecological and co-evolutionary adaptations between the parasites and their pollinators, as well as habitats. Stigma morphology is a highly informative taxonomic criterion that helps to resolve ambiguities in plant taxonomy and evolution and has proved to be a valuable complementary tool for Orobanchaceae species identification. It is noteworthy that this study supports the division between *Orobanche* and *Phelipanche*, as well as subgroups of stigma morphology corresponding to systematic and phylogenetic groups. Our research shows that the most useful features can be seen when observing the stigmas from the bottom side (previous research were related only to observations mainly from the front view), an aspect which has not been studied before. A comprehensive survey of the general and species of holoparasitic Orobanchaceae may lead to a better understanding of the floral morphology of the family.
